# On a New Method of Preserving Anatomical and Pathological Specimens

**Published:** 1854-07

**Authors:** John H. Brinton


### *On a new method of preserving Anatomical and Pathological Specimens.* By John H. Brinton, M. D.

The preservation of the animal tissues, in such manner as to present unchanged, their normal and abnormal relations, has ever been an object of study and interest to the practical Anatomist, the Pathologist and the Surgeon. But as yet, all attempts to retain, in an unaltered state, the size, shape and color of parts, have to a certain degree been unsuccessful.

Anatomical objects have hitherto been preserved in one of two states, either wet or dried. Both of these methods are, however, inadequate; for if fresh animal tissues be immersed in alcohol, or any other antiseptic fluid, they become blanched in color, condensed in structure, and consequently modified in size and shape. At the same time, they present inconveniences for demonstration. The method of preparation by drying is open to even more serious objections, since the parts are so shrivelled and displaced as to convey but an imperfect idea of their primitive relations.

Now, since this shrinking of the tissues and their decomposition, depend most probably upon atmospheric influence, it recently occurred to me, that should I be able so to exclude the air, as to cause all evaporation to cease, I would effectually prevent, not only the desiccation of the part, but also its decomposition. Acting upon this idea, I commenced a series of investigations; the success attendant upon which, up to the present time, has led me to submit the results to the profession.

My object being to encase hermetically every portion of the specimens, I selected for my earlier experiments a solution of gun cotton in ether, the ordinary collodion. By means of a brush, I applied this carefully upon every portion of the external surface of the object to be preserved. The ether quickly evaporating, a thin pellicle of the cotton was left, coating the entire preparation. This process was then repeated, and the preparation finished by the application of successive layers of damarra, copal and shellac varnishes.

But it early became evident to me, that collodion was entirely unfitted for the preservation of the generality of specimens, especially for those of any size and bulk. It possesses too slight a degree of tenacity, and is liable to become fissured, and to chip off; at the same time its tendency to form white opaque layers, when moisture is present, renders it unsuitable as a transparent coating. I was, therefore, obliged to make use of some other protective material, and for this purpose I selected gutta percha. This I dissolved in benzole, adding at the same time to the solution a few grains of caoutchouc in order to increase the elasticity of the pellicle. By filtering the viscid dark-colored result through animal charcoal a perfectly colorless solution may be obtained, which upon application deposits a tenacious film.

This film, unlike that left by the evaporation of the ethereal portion of the collodion, evinces but little tendency to opacity; and, indeed, for all practical purposes, may be said to be perfectly transparent. The tenacity of the thinnest pellicle is very great; it possess sufficient elasticity, is not liable to crack, and thus far has proven amply sufficient to prevent the occurrence of evaporation, shrinking and decomposition. At the same time, by repeated applications of the solution, the coating of gutta percha can be increased to any desirable thickness.

To prepare a muscular specimen, as, for example, one exhibiting the relations of the arm and forearm, a limb should be selected which has been previously injected with the chloride of zinc, arsenic or other antiseptic solution. When the parts have been sufficiently exposed by dissection, the whole limb should be coated with the colorless solution of gutta percha: a transparent pellicle will then be left by the evaporation of the benzole. This process should be repeated until a layer of considerable thickness is obtained. Upon the muscular mass, the gutta percha may be applied in much greater quantity. Should opacity here result it matters little, as in consequence of the blanching of the muscle, dependent upon the previous antiseptic injection, an artificial coloring process will be necessitated. In preparations, however, of perfectly fresh muscles, or of those which have been injected with Horner's solution, this will not be the case. The layers of gutta percha having been obtained of sufficient thickness, it will be found desirable to apply next a coating of collodion, which has been filtered, and then mixed with Venice turpentine. This preparation possesses no contractile powers whatever, but is of great body and consistency. It splints, as it were, the specimen securely, and ensures stability and firmness.

In order to impart a proper hue to those muscles, whose color may have been affected by the preceding processes, I make use of collodion stained with the wood of the Pterocarpus santalinus, (the ordinary red saunders.) The resulting color, when varnished, simulates closely that of fresh muscles. The specimen should then be completed by the application of damarra and copal varnishes. The adipose tissues, the tendons, fasciæ, nervous and cutaneous surfaces will present almost the appearance of a recent dissection; the muscle alone will possess an artificial color, and even this can be avoided.

Each specimen should be mounted on a board separately, and I have found the most convenient method for so doing, to consist in the preparation first of its dorsal surface. The object should then be placed upon the board; when the anterior surface, that intended for inspection and exhibition, can be finished in situ. I have also found it advisable to keep them covered by glass cases. The period required for the preparation and mounting of an object by the above process does not exceed five days.

I have now in my possession specimens of meat which have been preserved by the preceding method sixty days, without having been previously saturated by any antiseptic fluid. In one preparation which I examined, after removing the gutta percha coat at the expiration of forty-five days from its completion, no decomposition whatever had taken place. The fibres of the muscle were, however, somewhat blanched, and afforded a slight odor of the benzole used in the preparation. Exposed to the air, decomposition ensued at the expiration of four or five days.

I have prepared, in a similar manner, a number of specimens, not only of muscular, but also of nervous tissues, as the brain and spinal marrow. In these no shrinking has occurred, and no evidence of decomposition exists. On the contrary, the preparations now present a harder, firmer and more natural appearance, than at the date of their completion. In the nervous preparations the natural color is retained, and is visible through the transparent coatings. I am at present engaged in making application of the process to the preservation of pathological tissues, and with every prospect of success. I believe also that botanical specimens may be preserved in a similar manner, and, indeed, it seems to me not impossible, that, at some future day, an extension of this method may be rendered subservient to the preservation of meats, and all fresh animal tissues.

A longer period than has as yet elapsed, is required, of course, to test fully the value of the above proceeding; at the same time the results already obtained seem to me so satisfactory, as to warrant me in laying them before the profession.

